# Investigating opioid-related fatalities in southern Sweden: contact with care-providing authorities and comparison of substances

**DOI:** 10.1186/s12954-019-0354-y

**Published:** 2020-01-09

**Authors:** Lisa Andersson, Anders Håkansson, Peter Krantz, Björn Johnson

**Affiliations:** 1 Department of Social Work, Faculty of Health and Society, Malmö University, Malmö, Sweden; 2 Faculty of Medicine, Department of Clinical Sciences Lund, Psychiatry, Lund University, Lund, Sweden; 3 Malmö Addiction Centre, Region Skåne, Malmö, Sweden; 4 Faculty of Medicine, Department of Clinical Sciences Lund, Forensic Medicine, Lund University, Lund, Sweden

**Keywords:** Opioids, Opioid intoxication, Opioid-related mortality, Interventions, Care-providing authorities, Harm reduction

## Abstract

**Background:**

Opioid-related deaths have increased in Western countries over recent decades. Despite numerous studies investigating opioid-related mortality, only a few have focused on the lives of the deceased individuals prior to their deaths, specifically regarding contact with care-providing authorities such as health, social and correctional services. Furthermore, a change has been noted in the last two decades as to which opioids cause most deaths, from heroin to prescription opioids. However, studies comparing fatalities caused by different substances are rare. The aim of this study was to investigate contact with care-providing authorities during the year prior to death among individuals who died as a result of opioid intoxication and to analyse differences relating to which opioids caused their deaths.

**Methods:**

The study is based on retrospective register data and includes 180 individuals with a history of illicit drug use, who died from opioid intoxication in Skåne, Sweden, between 1 January 2012 to 31 December 2013 and 1 July 2014 to 30 June 2016. Intoxications caused by heroin, methadone, buprenorphine and fentanyl were included. Data were collected from the National Board of Forensic Medicine, regional health care services, municipal social services and the Prison and Probation Service. Statistical testing was performed using Pearson’s chi-square test, Fisher’s exact test and the Mann-Whitney *U* test to analyse group differences.

**Results:**

A total of 89% of the deceased individuals had been in contact with one or more of the care-providing authorities during the year prior to death; 75% had been in contact with health care, 69% with the social services, 28% with the Prison and Probation Service, and 23% had been enrolled in opioid substitution treatment at some point during their final year of life. Few differences appeared between the substance groups with regard to which opioid contributed to the death. In addition to opioids, sedatives were present in more than 80% of the cases. Individuals whose deaths were buprenorphine-related had been in contact with the social services to a significantly lesser extent during the year prior to death.

**Conclusions:**

The studied population is characterised by extensive contact with care-providing authorities, thus providing numerous opportunities for authorities to reach this group with preventive and other interventions. Few differences emerged between groups with regard to which opioid had contributed to the death.

## Introduction

Opioid-related mortality is an extensive and increasing global problem, which mainly affects people under the age of 50. Since the turn of the millennium, an increase in opioid-related deaths has been noted in several parts of the Western world [1–4]. According to international statistics, Sweden is among the European countries with the highest drug-related mortality [4, 5]. Drug-related deaths in Sweden have increased from 7.3 per 100,000 inhabitants in 2006 to 11.6 per 100,000 in 2017 [6]. About 80% of these deaths are opioid-related [4].

During the 2000s, there has also been an increase in the number of studies investigating opioid-related mortality in Western countries [7, 8]. There are currently a large number of studies based on demographic, forensic and medical data [9–21]. Several important risk factors for opioid-related mortality have been identified. Intravenous injection [22], poly-drug use [8, 23], non-participation in opioid substitution treatment (OST) [24], resuming non-medical use after a period of abstinence, discontinued treatment or release from prison [8, 24, 25] and a vulnerable social situation, e.g. homelessness [23, 26–29], all increase the risk of fatal intoxication among opioid-dependent people.

However, less is known about the lives of the deceased individuals during the period preceding their deaths—what contact they have had with authorities such as health care, social and correctional services, i.e. the authorities that offer different types of intervention with regard to problematic substance use. Contact with authorities has been highlighted in a small number of studies [13, 30, 31]. These studies indicate that people whose deaths are drug-related have generally been in contact with a range of care-providing authorities during the year prior to death.

In parallel with the increase in opioid-related deaths, there has also been a gradual change in many Western countries over the last two decades with regard to which opioids are implicated in the most deaths, from heroin to prescription opioids (analgesics such as fentanyl and oxycodone, but also substances used in OST, e.g. methadone and buprenorphine) and new psychoactive substances, mainly in the form of fentanyl analogs [1, 5, 14, 16, 19, 32–35]. In several countries, methadone overdoses have been a leading cause of death among opioid-dependent individuals [14, 36–39], which has been linked to the discussion on the diversion of medications from OST [40].

Previous studies that have analysed differences between deceased individuals in relation to which opioid they died from have mainly focused on medico-legal and demographic factors [11, 14, 16, 21]. Some differences appear in these studies, e.g. regarding the gender and age of those whose deaths were related to heroin and prescription opioids, respectively. However, there is no research showing whether there may be other differences, for example regarding the individuals’ social situation and contact with the health care system or the social services.

Thus, more research is needed regarding the extent to which people whose deaths are opioid-related are known to various authorities and to what extent they have been in contact with various welfare institutions prior to their deaths. Of particular interest is whether the deceased individuals have had experience of interventions aimed at opioid dependence, e.g. OST. There is also a need for further comparisons between deaths caused by different opioids. Such research should include both medico-legal and socio-demographic data, as well as information on contact with care-providing authorities, in order to be able to design adequate preventive measures and care interventions. The Nordic welfare states are well suited to such studies since they provide access to extensive public registers, from which data can be linked via personal identification numbers [41].

In this study, we investigate fatal opioid intoxications in southern Sweden among people with a history of illicit drug use. The purpose of the article is (1) to survey the deceased individuals’ contact with care-providing authorities during the year prior to death and (2) to analyse differences in their clinical picture, relating to which opioids caused their deaths.

## Data and method

### Setting and design

This is a retrospective register study based on an extensive data set from a research project on opioid-related deaths in Skåne (Scania), a county in the south of Sweden with approximately 1.36 million inhabitants. The larger project included individuals who died in Skåne during a period of 4 years, where any specified opioid was detected during forensic autopsy, irrespective of the cause of death. The data set covers two different time periods, 1 January 2012 to 31 December 2013 and 1 July 2014 to 30 June 2016, respectively. The use of these periods is due to the fact that in another sub-project, we use this data to compare deaths that occurred during two different periods, before and after a reform on patient choice in substitution treatment was introduced in Skåne. This reform has led to an increase in the availability of substitution treatment [42]. To identify deaths where opioids were detected during autopsy, a review was carried out of medical records at the Swedish National Board of Forensic Medicine in Lund, Sweden. The review covered all individuals registered as residing in Skåne at the time of their death and who died in Skåne. The review was carried out manually by the first author (LA), with the assistance of PK, in order to minimise the risk of missing cases. Opioids were detected in 503 of the approximately 4000 deaths, which were examined during the study’s two observation periods.^1^ The project was limited to individuals < 65 years of age. This meant that 116 individuals were excluded at an early stage.^2^

More than 90,000 deaths occur annually in Sweden, of which about 6% are subject to forensic autopsy. A forensic autopsy, including toxicological analysis, is carried out on behalf of the police or prosecutor when the cause of death is unknown or when there are unclear circumstances surrounding the death [44, 45]. This procedure takes place in more than 90% of drug-related deaths [46, 47].

### Study inclusion

In this study, we analyse deaths where the deceased individuals had a history of illicit drug use and the cause of death was intoxication, drawn from the larger data set described above. Intoxications caused by heroin, methadone, buprenorphine and fentanyl were included in the study. Other opioids were present in too few cases per substance to be included in comparisons.^3^

The classification regarding the history of illicit drug use in forensic records has been based on whether information on illicit drug problems was mentioned in the autopsy report or police investigation, whether the presence of injection marks had been noted in the autopsy report or whether illegal drugs (heroin/6-MAM, amphetamine, cocaine or THC) were detected in forensic analysis.

To determine whether a death was caused by intoxication, we have consulted the death certificates of the deceased individuals. The aim was to determine the extent to which intake of heroin, methadone, buprenorphine or fentanyl had been the direct cause of, or had essentially contributed to the death. In most cases, only one opioid was deemed to be of significance to the death. In two cases, the cause of death was caused by a combination of methadone and buprenorphine, and these cases were therefore excluded.

**Fig. 1:**
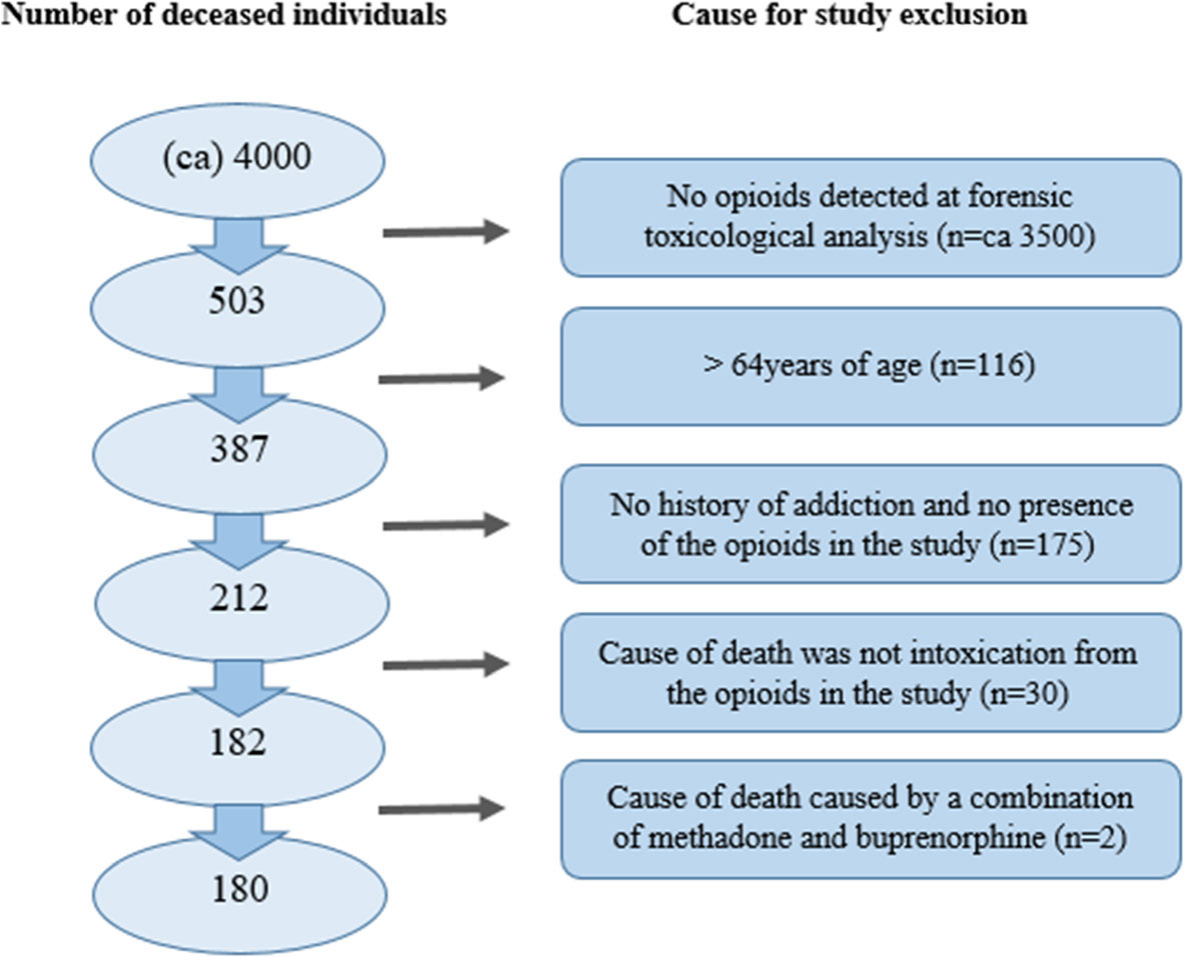
Study exclusion criteria. Opioids investigated in the study were heroin, methadone, buprenorphine and fentanyl

Based on the inclusion and exclusion criteria, the number of deaths due to intoxication from heroin, methadone, buprenorphine or fentanyl was 180, as shown in Table 1. Figure 1 shows the exclusion process of the participants in the study.

**Table 1:** Number of deaths due to intoxication in the study population distributed among substance groups

Substance	*n* (%)
Heroin	40 (22.2)
Methadone	82 (45.6)
Buprenorphine	30 (16.7)
Fentanyl^a,b^	28 (15.6)
Total	180 (100)

^a^Including seven cases where fentanyl findings consisted of acrylfentanyl, acetylfentanyl or furanylfentanyl

^b^Fentanyl is prescribed as patches, tablets and nasal spray in Sweden

### Data sources and variables

Data were collected from the Swedish National Board of Forensic Medicine, regional health care services (including OST), municipal social services and the national Prison and Probation Service. Information from the different authorities was collected through manual journal review (the National Board of Forensic Medicine, social services and partially health care) and from searches based on personal identity numbers in databases (the Prison and Probation Service and partially health care). The manual searches were performed by the first author (LA) and a medical student. The health care services, social services and the Prison and Probation Service will hereafter be referred to as “care-providing authorities”.

All data sources were linked via the personal identification number of the deceased, which is unique to every Swedish resident [41]. Unless otherwise reported, all variables are dichotomous with the variable values “yes” and “no”.

#### The National Board of Forensic Medicine

The data from the Swedish National Board of Forensic Medicine include demographic information about the deceased individuals’ gender, age, place of residence and place of death. The toxicological data indicate the presence and quantity of pharmaceutical drugs, alcohol and illegal substances. The data also include police reports and in some cases copies of health care records.

The construct “z-drugs” refers to the presence of zopiclone (zolpidem was not present in the data). “Sedatives” include the presence of at least one benzodiazepine, z-drug or pregabalin. “CNS stimulants” refer to the presence of amphetamine, cocaine, MDMA or methylphenidate. “Antidepressants” include the presence of prescription antidepressant medications. “Opioid only” refers to cases of poisoning from only one opioid, without the presence of other pharmaceutical drugs, alcohol or illegal substances. “Alcohol (> 0.5‰)” indicates that only cases with a blood alcohol concentration of > 0.5‰ are included, to avoid the risk of including cases of alcohol being produced in the body after death [48, 49]. In addition to toxicological data, we use the following variables, based on information from the Swedish National Board of Forensic Medicine’s records: “needle marks”, “stable housing” (refers to own housing, sub-tenancy housing, stable accommodation or housing with support and service for individuals with certain functional impairments) and “intoxication occurred in a private residence” including the individual’s own or someone else’s residence (intoxication outside private residences usually occurred in a public environment, in a hotel, treatment center or homeless shelter).

#### Regional health care

Health care is organised regionally in Sweden. There is one central body for the health care system in Skåne, regardless of whether the care is provided publicly or privately.^4^ Events that occurred in connection with death were not included in the data of the present study. Primary care was not covered by the review of medical records.

The following variables are used in this study: “health care contact” refers to contact with hospital care or OST during the year prior to death. “Inpatient health care” refers to inpatient physical health care or psychiatric/addiction health care. “Contact physical health care” refers to contacts irrespective of whether they involved inpatient or emergency care, while “inpatient physical health care” only includes inpatient care. “Contact psychiatric/addiction health care” refers to contacts regardless of whether they involved inpatient or emergency care. “Inpatient psychiatric/addiction health care” refers only to inpatient care. “Attempted suicide” is based on texts in medical records that indicate a suicide attempt during the year prior to death. “Intoxication” is based on ICD codes for intoxication in medical records. “Opioid substitution treatment” refers to ongoing or discontinued OST during the year prior to death. Buprenorphine is recommended as the first-line choice of medication in OST in Sweden [50] and is usually prescribed in combination with naloxone.

#### Municipal social services

In Sweden, municipal social services have responsibility for providing non-medical treatment and social support for people with misuse and dependence related to drugs or alcohol.^5^ Skåne comprises 33 municipalities, each of which has its own social services administration and which maintains separate registers. People with problematic substance use may receive help in the form of both outpatient care and residential rehabilitation at treatment facilities following a structured care program. Treatment in full-day care in residential treatment facilities usually takes place on a voluntary basis, after the person has sought treatment from his or her municipality’s social services. However, there is also the possibility of being subjected to compulsory care, which may be relevant in cases of severe addiction problems where serious medical or social complications are likely and if the person does not consent to care on a voluntary basis. Compulsory care may be applied in relation to people aged 18 and over for a maximum period of 6 months.^6^ Compulsory care accounts for approximately 15% of the total amount of residential treatment provision [51]. The social services also administer the provision of financial assistance to those who lack the means to support themselves.

Regarding contact with the social services, the following variables have been included in the study: “social services contact” refers to contact concerning addiction problems and/or financial assistance during the year prior to death. “Addiction unit” refers to ongoing or completed contact with the social services addiction unit during the year prior to death. “Voluntary care” refers to ongoing or completed voluntary care for addiction problems during the year prior to death. “Compulsory care investigation” refers to whether an investigation in accordance with compulsory care legislation was initiated during the year prior to death. “Compulsory care” refers to having received care in accordance with the compulsory care legislation during the year prior to death, including ongoing care.

#### The Swedish Prison and Probation Service

The Swedish Prison and Probation Service is the national authority responsible for enforcing prison and community supervision sentences. Both in prison and while under supervision, the service provides a number of rehabilitation programs addressing drug addiction.

Two variables have been coded in relation to experiences of the Prison and Probation Service: “supervision” refers to ongoing or completed supervision via the probation service during the year prior to death, either in the form of probation or upon conditional release from prison, while “prison” refers to whether the person served a prison sentence during the year prior to death.

### Statistical analysis

The data were analysed by means of frequency calculations, cross-tabulations and comparisons of medians. Significance testing of group differences has been carried out using Pearson’s chi-square and Fisher’s exact test. The Mann-Whitney *U* test for independent samples was used to compare medians between groups. Methadone, the most common opioid among the deceased, is the reference value for pairwise comparisons between the substance groups. All analyses were performed using IBM SPSS Statistics version 24 for Windows. A *p* value < 0.05 was considered statistically significant.

## Results

### Description of demographic and forensic data

Table 2 presents the study population (*n* = 180) on the basis of demographic variables and forensic data. The presentation is grouped based on which opioid was dominant in the death. The majority of the deceased were men (82%), and the median age was 34.5 years. Just over two-thirds (69%) had a stable housing situation, and most incidents of intoxication (81%) occurred in a private residence.

**Table 2:** Sociodemographic characteristics, substance-related data and forensic data among the study population. Percentages

	Methadone (*n* = 82) (reference)	Buprenorphine (*n* = 30)	Heroin (*n* = 40)	Fentanyl (*n* = 28)	Total (*n* = 180)
Age at death, median (Q1; Q3)^a^	34.5 (25.75; 43.25)	30.5 (26.0; 38.0)	35.0 (27.25; 44.50)	35.5 (27.5; 46.0)	34.5 (26.25; 42.75)
Gender (male)	82.9	86.7	85.0	71.4	82.2
Stable housing	70.7	76.7	65.0	64.3	69.4
Intoxication occurred in a private residence	81.7	93.3	72.5	75.0	80.6
Needle marks	31.7	30.0	97.5***	39.3	47.2
Alcohol (> 0.5‰)^b^	9.8	16.7	37.5***	10.7	17.2
Benzodiazepines	74.4	86.7	67.5	53.6*	71.7
Z-drugs	15.9	26.7	22.5	28.6	21.1
Pregabalin	22.0	36.7	17.5	32.1	25.0
Sedatives	82.9	93.3	82.5	71.4	82.8
THC	19.5	23.3	30.0	7.1	20.6
CNS stimulants	26.5	10.0	32.5	17.9	23.3
Opioid only^c^	3.7	0.0	5.0	25.0*	6.7
Antidepressants	18.3	40.0*	32.5	14.3	24.4

Methadone is the reference value for *x*^2^ comparisons between the substances

*p value of < 0.05; ***p value of < 0.001

^a^Independent samples Mann-Whitney *U* test used to compare medians between groups

^b^Two cases were removed because the analyses had not been carried out on femoral blood. Both these cases were methadone intoxications

^c^Fisher’s exact test was used for this variable

A large majority of the deaths involved intoxication via a combination of illegal drugs and pharmaceuticals, and other substances were present, in addition to opioids, in most cases. The most common substance group was benzodiazepines, which were found in 72% of the deceased individuals. Pregabalin (25%), antidepressant drugs (24%), alcohol > 0.5‰ (21%) and Z-drugs (21%) were also common findings. A total of 83% had a sedative substance in the form of benzodiazepines, Z-drugs or pregabalin in the body at the time of death. THC and central nervous system stimulants were found in 21% and 23% of deaths, respectively.

In pairwise comparisons between methadone and the other investigated opioids, few significant differences emerged. As regards the presence of other substances, significant differences were identified in relation to alcohol, benzodiazepines and antidepressants. Compared with methadone-related deaths, alcohol was present to a significantly greater extent in heroin-related deaths (*p* < 0.001), benzodiazepines occurred at significantly lower rates among deaths that were fentanyl-related (*p* = 0.04) and the presence of antidepressants was significantly more common among buprenorphine-related deaths (*p* = 0.02). Deaths without the simultaneous occurrence of other substances (pharmaceutical drugs, alcohol or illegal substances) were significantly more common among the fentanyl-related deaths than among the methadone-related deaths (*p* = 0.02).

The presence of needle marks differed in relation to deaths caused by methadone and heroin, respectively. Deaths caused by heroin were significantly more likely to be associated with the presence of needle marks than methadone-related deaths (*p* < 0.001).

### Contact with health care

The deceased individuals had numerous contacts with health care services during the year prior to death, as shown in Table 3. Here, contact with the health care services relates to OST, inpatient care and emergency care in hospital. Of the study population, 75% had been in contact with the health care services, with non-significant differences between the substance groups. A total of 51% had received inpatient treatment, 32% had been admitted to hospital following intoxication during the year prior to death and 11% had been admitted to hospital following an attempted suicide.

**Table 3:** Contact with health care services during the year prior to death. Percentages

	Methadone (*n* = 82) (reference)	Buprenorphine (*n* = 30)	Heroin (*n* = 40	Fentanyl (*n* = 28)	Total (*n* = 180)
Health care contact	73.2	73.3	77.5	78.6	75.0
Inpatient health care	54.9	40.0	50.0	53.6	51.1
Physical health care	52.4	53.3	57.5	69.7	56.1
Inpatient physical health care	36.6	33.3	30.0	46.4	36.1
Psychiatric/addiction health care	39.0	50.0	50.0	46.4	44.4
Inpatient psychiatric/addiction health care	32.9	23.3	35.0	32.1	31.7
Opioid substitution treatment	32.9	16.7	17.5	10.7*	23.3
Previous intoxication	26.8	40.0	35.0	35.7	32.2
Attempted suicide^b^	11.0	20.0	10.0	0.0	10.6

Methadone is the reference value for *x*^2^ comparisons between the substances.

*p value of < 0.05

^b^Fisher’s exact test used for this variable

Of the recorded health care variables, the only difference found between the substance groups was related to OST during the year prior to death. Those who died due to fentanyl were significantly less likely to have encountered this form of health care provistion than those whose deaths were methadone-related (*p* = 0.023).

### Contact with social services and the Prison and Probation Service

Table 4 shows that 69% of the deceased had been in contact with the social services during the year prior to death. In total, 57% had been in contact with the social services’ addiction unit and 48% had an ongoing or completed a voluntary addiction treatment intervention or were housed by the social services in the year preceding death. An investigation in accordance with compulsory care legislation had been initiated in 15% of cases in the last year of life, and 6% had been subjected to compulsory care. Significant differences were found with regard to contact with the social services during the year prior to death, with the group of buprenorphine-related deaths having significantly fewer such contacts than the methadone-related deaths, both in terms of contact solely with the addiction unit (*p* = 0.048) and contact with addiction or financial assistance units (*p* = 0.02).

**Table 4:** Contact with social services and the Prison and Probation Service during the year prior to death. Percentages

	Methadone (*n* = 82) (reference)	Buprenorphine (*n* = 30)	Heroin (*n* = 40)	Fentanyl (*n* = 28)	Total (*n* = 180)
Social services contact	73.2	50.0*	75.0	71.4	69.4
Addiction unit	61.0	40.4*	60.0	60.7	57.2
Voluntary care	52.4	33.3	47.5	50.0	47.8
Compulsory care investigation	17.1	6.7	17.5	14.3	15.0
Compulsory care^b^	6.1	0.0	5.0	10.7	5.6
Supervision	26.8	30.0	30.0	25.5	27.8
Prison^b^	4.9	6.7	12.5	10.7	7.8
Contact with one or more authorities	89.0	86.7	90.0	92.9	89.4

Methadone is the reference value for *x*^2^ comparisons between the substances

*p value of < 0.05

^b^Fisher’s exact test used for this variable

The data from the Prison and Probation Service indicate that 28% of the deceased had been under supervision during the year prior to death and 8% had served a prison sentence. There were no significant differences between the substance groups.

Table 4 also shows that 89% of the deceased had been in contact with at least one of the three main care-providing authorities for individuals with addiction problems—health care, social services or the Prison and Probation Service—during the year prior to death.

## Discussion

In this study, we have investigated 180 opioid-related deaths where the cause of death was intoxication due to the intake of heroin, methadone, buprenorphine or fentanyl. We found that the deceased had been in contact with the investigated care-providing authorities to a substantial extent during the year prior to death and that few differences emerged between groups with regard to which opioid had contributed to the death.

### A group with high levels of contact with care-providing authorities

The individuals who died of opioid-related intoxications had a variety of problems and were characterised by extensive contact with care-providing authorities. Nine out of ten had been in contact with one or more of the three authorities included in the study during the year prior to death. They were thus largely known to the health care and social services and (to a lesser extent) the Prison and Probation Service. This is in line with the results of other studies, which have highlighted contact with care-providing authorities among people whose deaths were drug-related [13, 30, 31]. In a Norwegian survey, Gjersing et al. found that just over 80% of people whose death was drug-induced were known to the authorities during the year prior to death [13], and in a Scottish study, over 70% of the study population had been in contact with authorities within 6 months preceding death [31].

The results indicate that there are considerable opportunities for society’s care-providing authorities to reach people with risky opioid use in order to provide interventions to prevent death from intoxication. A review and improvement of prevention measures is necessary, as has previously been suggested in other studies using similar approaches [13, 30, 31]. Access to harm reduction interventions for people who use opioids has increased in Sweden in recent years, but remains low in comparison with many other Western European countries [4]. In order to reach their target group, such interventions would need to be developed and made available via several facilities and services which come into contact with opioid users.

A large majority (82%) of the deceased in the current study were men, and the median age was 34.5 years, figures that are in line with other studies on drug-related deaths [2, 14, 18, 52]. A total of 70% of the population had a stable housing situation at the time of death. Most of the deaths, approximately 80%, occurred in a private residence belonging to the deceased or another person. Other studies have also found that the majority of drug-related deaths occur in private homes and that only a minor proportion occur in public environments [16, 18, 20, 30, 53]. Public drug-consumption rooms are currently not available in Sweden.

Over 80% of the deceased had some form of sedative drug (benzodiazepines, Z-drugs or pregabalin) in the body at the time of death, in addition to opioids. The concomitant use of these substances constitutes a risk factor for both fatal and non-fatal overdose [54, 55]. Benzodiazepines were the most common and were found in 72% of the deaths. Similar results regarding the presence of other drugs, mainly benzodiazepines, have been demonstrated in some previous studies [13, 21, 54], while other studies have presented significantly lower figures for benzodiazepines [11, 16, 19]. One way to prevent such intoxications might be to encourage physicians to reduce their prescriptions of benzodiazepines and other sedatives to opioid users. The mandatory use of prescription drug monitoring programs might serve as a means of limiting such risky prescription practices [17].

One third of the investigated subjects had been admitted to hospital following an overdose in the final year of life, which is suggestive of extensive and severe drug problems [56]. It also emerged that only one quarter of the study population were in OST at the time of death or had discontinued such treatment during the final year of life. We lack data on the extent to which the individuals in the study fulfilled the diagnostic criteria for opioid dependence, but it is likely that considerably more people could have been considered for OST than those who actually participated in such treatment during the year prior to death. Both access to OST and the retention rate in treatment have increased in Skåne in recent years [42], but this study indicates that there is still room for improvement.

Half of the people in the study had sought and been granted interventions from the social services for addiction treatment or housing during the year prior to death. In the event of severe addiction problems, and when the person is not interested in receiving care on a voluntary basis, compulsory care can be utilised for a maximum of 6 months. About 1 in 20 persons (6%) among the deceased was cared for based on the compulsory care legislation during the year prior to death. In an additional number of cases (15%), investigations were initiated on the basis of this legislation, but no decision on such care was taken. This result is not unexpected since the study population is comprised of a group to whom this legislation is addressed and who could probably have been subjected to compulsory care on the basis of the legislation to an even greater extent.

### Differences between deaths caused by the various substances investigated

Of the investigated deaths, nearly half, 46%, were caused by methadone. Heroin intoxications accounted for 22%, and the remaining deaths were evenly distributed between buprenorphine (17%) and fentanyl (16%), respectively. This is in line with previous Swedish studies [52, 57, 58], in which deaths related to methadone and buprenorphine in particular have increased sharply since 2005.

There were few significant differences between the various substances examined in the study with regard to either demographic or forensic variables, or contact with care-providing authorities. Yet, some differences were noted. To have been in OST during the final year of life was more common among the methadone-related deaths (33%) than among those whose deaths were fentanyl-related (11%). This is in line with other Swedish studies, which have shown that 75–80% of those who die as a result of methadone or buprenorphine do not have ongoing OST [14, 58, 59]. Previous research has noted that opioid-dependent people sometimes use illicit methadone or buprenorphine as a form of “self-treatment” in order to avoid injecting heroin [60, 61]. However, such non-prescribed use also involves substantial risks, as has been shown by this and other studies [15, 17, 36, 59]. Since non-prescribed use is often driven by pseudo-therapeutic motives [62], increased access to OST might decrease the illicit demand for these substances [60, 61].

The individuals who died of buprenorphine intoxication had been in contact with the social services with regard to both addiction problems and financial assistance to a much lesser extent than those who died of methadone intoxication. Among the buprenorphine-related deaths, no one had been subjected to compulsory care in the year prior to death. This may indicate that those who died of buprenorphine intoxication had less severe substance use problems and were somewhat less “experienced” with regard to interventions aimed at the substance use. It can be noted that no one had died due to the intake of buprenorphine alone, in contrast to the other substances, where mono-substance deaths did occur. Deaths due to buprenorphine alone, without the simultaneous presence of benzodiazepines or other depressant substances, are generally very rare due to the protective “ceiling effect” of buprenorphine on respiratory depression [24, 54, 63]. Thus, this further highlights the need to prevent poly-substance use, such as use of sedatives, in individuals misusing opioids including buprenorphine.

Fentanyl is an extremely potent opioid compared to the other substances included in the study—50 to 100 times more potent than morphine [64]. In this study, there was a significant difference in the presence of benzodiazepines between the fentanyl-related and the methadone-related deaths, with benzodiazepines being present to a lesser extent in connection with fentanyl death. Compared with methadone, fentanyl-related deaths also occurred significantly more frequently without the involvement of sedatives, alcohol or illegal substances. These results underline the risk of fatal intoxication when using fentanyl alone.

The listing of needle marks in forensic protocols was significantly more prevalent among heroin-related fatalities compared to those related to methadone. This is an expected result, partly because injection is the most common method of intake for people who use heroin and partly because the injection of heroin leads to overdose to a greater extent than smoking (inhalation) [65]. The result that needle marks only occurred in 30–40% of those who died from substances other than heroin might be an indication that parts of the population have made active attempts to avoid overdoses and other negative consequences of injecting [61]. An additional interpretation may be that the method of intake is adapted to the substance that is available at the time. However, this finding demonstrates the high risk of overdose death also with non-injecting routes of administration.

Results from research comparing deaths caused by different opioids have been ambiguous. One problem that complicates comparisons between studies is that the substances are clustered in different ways. Often, prescription opioids other than methadone are presented as a single group, and few studies have buprenorphine listed as a separate substance group. In a Swedish study by Wikner et al., individuals experiencing buprenorphine deaths were significantly younger than those who experienced methadone deaths (28 compared to 35.5 years) [14]. In other studies, those whose deaths were caused by heroin or fentanyl were younger in comparison to those whose deaths resulted from prescription opioids [21] or non-heroin opioids [16]. In our study, however, no significant differences emerged between the substance groups with regard to median age.

In a previous study, Nechuta et al. found an association between alcohol and an increase in the odds for heroin overdose deaths in comparison with other opioid deaths, which supports our study’s finding that alcohol was present to a significantly greater extent in heroin fatalities than in methadone fatalities [21]. Visconti et al., however, did not find alcohol to be significantly associated with heroin compared to non-heroin opioid deaths [16].

The few significant differences in the clinical background of the deceased individuals in this study suggest that they constitute a relatively homogeneous group, based on which opioid caused the death. However, there are two substance-specific aspects that may be of importance when adapting preventive interventions. One relates to the use of counselling that highlights the risks specifically associated with non-injection opioid use, based on the finding that the majority of the deceased lacked needle marks. The second concerns the targeting of preventive measures to reduce alcohol use among heroin users, since the co-occurrence of alcohol was particularly high among the heroin-related deaths.

### Strengths and limitations

Potential strengths of the current study include the fact that it covers a complete regional population, with no missing cases, and that the data are register based and thus linked at an individual level between the different data sources. The study also includes data obtained from social services, which has largely been lacking in previous research.

Among the limitations of the study can be mentioned the fact that additional data from other authorities could have been obtained in order to provide further in-depth knowledge of the studied population. This might, for example, have included demographic data concerning income and employment, relationship status and residential area, as well as additional information from the health care services regarding primary care contact, diagnoses and prescription history.

Other limitations are that the study covers relatively few cases, particularly with regard to the buprenorphine and fentanyl deaths, and is based on a limited, retrospective follow-up period of 1 year. With a larger study population, more significant differences between the substance groups might have emerged, for example regarding the buprenorphine-related deaths, where non-significant tendencies were noted in some comparisons.

## Conclusions

Developing a broader understanding of the lives and deaths of opioid users is essential for the development and provision of effective treatment and harm reduction interventions. In this study of 180 Swedish individuals whose deaths were caused by opioid intoxication, almost 90% were known to care-providing authorities. The group had largely received support and interventions from health care, the social services or the Prison and Probation Service during the final year of life. This implies a major potential for authorities to reach this group with preventive and harm-reducing interventions. A review and improvement of such interventions is necessary, however, to effectively target individuals at risk of opioid intoxication.

Few differences emerged between groups with regard to which opioid had contributed to the death. In this respect, the investigated population appears to comprise a relatively homogeneous group, with regard to both forensic and demographic data and also contact with and interventions from care-providing authorities during the year prior to death.

## Data Availability

The SPSS datasets used in the current study are not publicly available due to restrictions made by the Regional Ethical Review Board in Lund, Sweden, but are available from the corresponding author on reasonable request.

## Footnotes

1 Each year, around 1000 autopsies are carried out at the National Board of Forensic Medicine’s office in Lund. However, the geographical catchment area not only consists of Skåne but also includes neighboring provinces.

2 Previous Swedish studies have shown that fatal opioid overdoses due to heroin, methadone and buprenorphine are unusual among people aged > 64 years [14]. Furthermore, clinical populations of people with opioid dependence are rarely older than 65 years of age [43]. Sweden also has a high coverage of forensic autopsies carried out when cause of death is unclear; this includes elderly people who die outside of hospital. The 116 individuals aged > 64 years excluded from the larger research project mainly had presence of morphine/codeine, oxycodone or tramadol in their bodies, but these substances had (in most of cases) not contributed to their deaths.

3 Tramadol intoxication was the cause of death in 15 cases, oxycodone intoxication was the cause of death in 13 cases, morphine intoxication was the cause of death in 8 cases, codeine intoxication was the cause of death in 5 cases, dextropropoxyphene intoxication was the cause of death in 3 cases and ketobemidone and loperamide intoxication were each the cause of death in a single case. In these cases, no assessment has been made of the history of illicit drug use.

4 Health care in Sweden is tax-financed and heavily subsidised, with regard to both health care visits and medication. The cost to the patient of outpatient medical care is restricted to a maximum of SEK 1100 per year. The cost to the patient of inpatient care is restricted to a maximum of SEK 100 per day (depending on income), regardless of the extent of the health care. The cost to the patient of medicines is restricted to a maximum of SEK 2300 per year.

5 Care and treatment for addiction problems are tax-funded and in principle free of charge in Sweden. The cost to the patient of residential treatment via the social services is restricted to a maximum of SEK 100 per day (depending on income).

6 Compulsory care is regulated by the Swedish Act (1988: 870) on the Care of Addicts in Certain Cases.
